# *Serendipita indica* changes host sugar and defense status in *Arabidopsis thaliana*: cooperation or exploitation?

**DOI:** 10.1007/s00425-021-03587-3

**Published:** 2021-02-23

**Authors:** Michael W. Opitz, Roshanak Daneshkhah, Cindy Lorenz, Roland Ludwig, Siegrid Steinkellner, Krzysztof Wieczorek

**Affiliations:** 1 Department of Crop Sciences, Institute of Plant Protection, University of Natural Resources and Life Sciences, Tulln an der Donau, Austria; 2 Department of Food Sciences and Technology, Institute of Food Technology, University of Natural Resources and Life Sciences, Vienna, Austria

**Keywords:** Carbohydrates, Endophytic fungi, Invertases, *Piriformospora indica*, Plant defense, Sucrose synthases

## Abstract

**Main conclusion:**

Manipulation of sugar metabolism upon *S. indica* root colonization triggers changes in sugar pools and defense responses in *A. thaliana*.

**Abstract:**

*Serendipita indica* is an endophytic fungus that establishes mutualistic relationships with many different plants including important crops as well as the model plant *A. thaliana*. Successful root colonization typically results in growth promotion and enhanced tolerance against various biotic and abiotic stresses. The fungus delivers phosphorus to the host and receives in exchange carbohydrates. There are hints that *S. indica* prefers hexoses, glucose, and fructose, products of saccharose cleavage driven by invertases (INVs) and sucrose synthases (SUSs). Carbohydrate metabolism in this interaction, however, remains still widely unexplored. Therefore, in this work, the sugar pools as well as the expression of *SUS*s and cytosolic *INVs* in plants colonized by *S. indica* were analyzed. Using *sus1/2/3/4* and *cinv1/2* mutants the importance of these genes for the induction of growth promotion and proper root colonization was demonstrated. Furthermore, the expression of several defense-related marker genes in both multiple mutants in comparison to the wild-type plants was determined. Our results show that in colonized *A. thaliana* plants *S. indica* manipulates the sugar metabolism by altering the expression of host’s *INV* and *SUS* and modulates both the sugar pools and plant defense in its favor. We conclude that the interaction *A. thaliana*–*S. indica* is a balancing act between cooperation and exploitation, in which sugar metabolism plays a crucial role. Small changes in this mechanism can lead to severe disruption resulting in the lack of growth promotion or altered colonization rate.

**Supplementary Information:**

The online version contains supplementary material available at 10.1007/s00425-021-03587-3.

## Introduction

Beneficial plant–microbe interactions evolve since 400 million years and play until today an important role for most plant species or even whole ecosystems (Parniske 2008). The best-known microbial group establishing such associations are arbuscular mycorrhizal fungi (AMF). During root colonization, these obligate symbionts facilitate uptake of important minerals—especially phosphorus and nitrogen (Luginbuehl and Oldroyd 2017). Further, they can confer tolerance and resistance to different abiotic stresses (drought, salt, toxins, or heavy metals) and to pathogenic organisms. In return, these fungi receive up to 20% of the plants fixed carbohydrates, mainly in form of hexoses (Parniske 2008).

Next to AMF, in the last years the focus of research moved to a group of Sebacinales with two phylogenetic subgroups: Sebacinaceae and Serendipitaceae (Weiß et al. 2011). Serendipitaceae contains several species with *Serendipita indica* (formerly *Piriformospora indica*) being the best studied member. This root endophyte was originally isolated in the Indian Thar Dessert from a spore of the AMF *Funneliformis* (= *Glomus*) *mosseae* (Verma et al. 1998). The fungus is able to establish mutualistic relationships with many different plants (Franken 2012), including the model plant *Arabidopsis thaliana* (Peskan-Berghöfer et al. 2004). A successful root colonization is divided in four different stages: (1) extracellular; (2) biotrophic; (3) cell death-associated and (4) fungal reproduction (Jacobs et al. 2011), leading to increased plant growth and development (reviewed in Franken 2012) and enhanced biotic and abiotic stress tolerance (reviewed in Gill et al. 2016).

Although the interaction between *S. indica* and its plant partners is widely considered as mutualistic (Zuccaro et al. 2011), after successful overcoming the first physical barriers the fungus must cope, similar to pathogens, with basic plant defense responses. To do that, the endophyte suppresses or manipulates these initial defense as described by Jacobs et al. (2011) and subsequently grows intracellularly. In this phase of colonization, the mutualistic nutritional exchange between *S. indica* and the host plant can be established. For that purpose, *S. indica* grows intracellularly and eventually forms coiled structures in root cells similar to arbuscules of AMF (Schäfer and Kogel 2009). In these structures, a symbiotic interface is formed in the apoplastic space that involves the infolding of the periarbuscular membrane, which is continuously aligned with the plant plasma membrane of the cortical cell surrounding the fungal wall (Felle et al. 2009). At this interface, the nutrient exchange of phosphate and carbohydrates occurs (Shachar-Hill et al. 1995; Karandashov and Bucher 2005). These carbohydrates constitute the cost demanded by the fungus for delivering phosphates to the host (Bonfante and Anca 2009). The mechanism related to the carbohydrate and phosphate exchange across the symbiotic interface is still unknown. In the past, it could only be assumed that among different carbohydrates endophytic fungi like *S. indica* prefer simple sugars, glucose, and fructose, and their uptake is generally similar to AMF. This hypothesis is supported, e.g. by the presence of monosaccharide transporters GpMST1 (Schüssler et al. 2006) and GspMST2 (Helber et al. 2011) in glomeromycotan fungi. Both transporters were shown to play a pivotal role during the symbiotic root colonization. Furthermore, the mutualistic fungus *Phomopsis liquidambaris* was shown to prioritize host hexoses in both, in vitro culture assays and *in planta* (Sun et al. 2020). Moreover, Schäfer et al. (2009) showed lower hexose and starch contents in *S. indica* colonized barley roots and the work of Zuccaro et al. (2011) delivered another evidence revealing 19 putative hexose transporter genes in *S. indica* genome. Lahrmann et al. (2013) showed up-regulation of some of them during colonization of *A. thaliana* roots. More recently, Rani et al. (2016) characterized *PiHXT5* in more detail and showed its specific regulation during symbiosis with maize. Accordingly, Strehmel et al. (2016) made a confirming observation and demonstrated an increased concentration of different carbohydrates in *A. thaliana* roots colonized by *S. indica*.

In higher plants, sucrose is the main produced carbohydrate playing an important role in different biological processes (Salerno and Curatti 2003). It is transported through the phloem (Haritatos et al. 2000; Lemoine et al. 2013) and enters sink cells (e.g. roots) through plasmodesmata or the cell wall where it gets further processed (Wind et al. 2010). In *A. thaliana*, two types of transporter proteins, AtSUCs and AtSWEETs, actively facilitate this translocation (Durand et al. 2018). Once sucrose reaches its sink cell, sucrose synthases (SUSs), and invertases (INVs) split this disaccharide into its monomers glucose and fructose (Koch 2004). INVs cleave sucrose irreversibly, whereas SUSs produce fructose and UDP glucose, enabling reversible sucrose synthesis. In *A. thaliana*, six *AtSUS* (Baud et al. 2004; Bieniawska et al. 2007) and nine *AtINV* genes (Barratt et al. 2009; Barnes and Anderson 2018) are driving these reactions.

Besides their importance in plant nutrition, carbohydrates act as signaling molecules in many developmental processes as well as in defense responses (Wind et al. 2010). For instance, while sucrose mainly benefits cell differentiation and maturation, hexoses favor cell division and expansion (Koch 2004). Especially the role of sugar molecules in plant defense is recently getting increased attention (Bolouri Moghaddam and Van den Ende 2012; Tauzin and Giardina 2014; Trouvelot et al. 2014). In *A. thaliana*, Thibaud et al. (2004) showed increased *AtPR-2* gene expression, when sucrose was added to the in vitro growth medium. Gebauer et al. (2017) could show that increased sugar levels in *A. thaliana sweet11/sweet12* led to priming of the SA-pathway and therefore better protection against hemibiotroph fungus *Colletotrichum higginsianum*. These results are supported by work from Gómez-Ariza et al. (2007), who showed that higher sucrose levels in leaf tissue of transgenic rice plants trigger sucrose-dependent priming, resulting in a better protection against fungal and bacterial pathogens.

Recent reports indicate that *S. indica* takes up hexoses via functional transporters (Nizam et al. 2019). Accordingly, available sucrose in *A. thaliana* roots transported from the photosynthetic tissues must be further processed into hexoses. This can be achieved by shifts in the sugar pools and/or targeted manipulation of plant genes coding for sucrose-cleaving enzymes upon colonization. To test both possibilities, we performed sugar pool analyses of roots and shoots of *S. indica*-colonized plants. In the next step, we focused on host plant sucrose-cleaving enzymes, *SUCs* and *CINVs* and their expression during *S. indica* colonization of *A. thaliana*. Furthermore, to study whether these genes play an important role during successful colonization, we investigated quadruple mutant *sus1/2/3/4* and double mutant *cinv1/2* as well as analyzed the colonization rate and growth promotion of colonized plants. We also determined the expression of some important defense-related genes in both multiple mutants in comparison to the wild type plants. Our results demonstrate that in colonized *A. thaliana* plants, *S. indica* manipulates the sugar metabolism by altering the expression of host’s sucrose-processing enzymes and modulates both the sugar pools and plant defense clearly in its favor.

## Materials and methods

### Plant material and seed sterilization

All *A. thaliana* lines used in this work (Col-0 and T-DNA lines; Table 1) were kindly provided by Dr. Alison Smith (John Innes Centre, Norwich, UK). Sterilization of seeds was done according to Bohlmann and Wieczorek (2015). Briefly, seeds were surface-sterilized for 10 min in 5% Ca(OCl)_2_ with 0.1% Tween 20 followed by 5 min incubation in 70% ethanol and three subsequent washing steps in sterile dH_2_O.

**Table 1:** Plant material used in this work

Line name	Locus	Function	References
Col-0		Wildtype	
*sus1/2/3/4*	At5g20830/At5g49190/At4g02280/At3g43190	Sucrose synthase	Barratt et al. (2009)
*cinv1/2*	At1g35580/At4g09510	Cytosolic invertase	Barratt et al. (2009)

### Cultivation of *A. thaliana*

Plants were grown according to Sijmons et al. (1991) in sterile Petri dishes (9 cm diameter) on Knop medium in a culture room with a 16/8 light/dark photoperiod at 25 °C. Plates were prepared according to Cabello et al. (2014). Briefly, Knop medium of the shoot area (upper 1/3) contained 20 g L^−1^ sucrose (Knop+), whereas Knop medium of the root area (lower 2/3) contained no sucrose (Knop−). Each Petri dish contained on average 8–12 seeds. Petri dishes with plants harvested at earlier time point [3 day after inoculation (dai)] contained more seeds (12 seeds) than Petri dishes with plants harvested at later time points (7–14 dai: 8–10 seeds). Plates were sealed with parafilm and put into a culture room at the above-mentioned conditions.

### *S. indica* cultivation and fungus inoculation

*Serendipita indica* was kindly provided by Prof. Ralf Oelmüller (Department of Plant Physiology, Matthias Schleiden Institute of Genetics, Bioinformatics and Molecular Botany, Friedrich-Schiller-University Jena, Jena, Germany). The fungus was stock-cultured on Käfer’s medium with adaptions from Johnson et al. (2011). Briefly, the fungus was precultured every four weeks by transferring fungal plugs on new Petri dishes containing Käfer’s medium. Every 6–12 months, the fungus was co-cultivated and re-isolated from *A. thaliana* roots. Experimental plates were inoculated with *S. indica* plugs of 6 mm diameter excised from 4-week-old *S. indica* culture grown on Käfer’s medium. Two plugs were placed top side down (for immediate contact with the new medium) in the root area next to the root tips. Control plates were inoculated with the same number of Käfer’s medium plugs without a fungus.

### Harvesting and processing of plant material

Root and shoot material of colonized roots and shoots as well as their respective noncolonized controls were harvested 3, 7, and 14 dai. The collected material was immediately flash frozen in liquid nitrogen to prevent enzymatic or transcriptional alterations. Harvested plant material was ground with a Mixer Mill MM 400 (Retsch GmbH, Haan, Germany) for 1–3 min at 30 Hz into fine powder. Subsequently, the samples were stored at − 80 °C until further use.

### Sugar pool analysis

#### Extraction

For carbohydrate extraction, roots and shoots of *A. thaliana* Col-0 colonized by *S. indica* as well as their noncolonized controls were harvested, ground, and stored as described above. Soluble carbohydrate extraction was performed according to Leach and Braun (2016) with adaptions from Srivastava et al. (2008). Briefly, samples were immersed in ice-cold MCW-extraction solution (methanol:chloroform:water, 12:5:3, by vol.). Samples consisted of 30–135 mg of fine ground frozen plant tissue. Subsequently, 10 × volume of ice-cold MCW extraction solution was added. Samples were vortexed and extraction was performed at 50 °C for 15 min using a thermomixer (Eppendorf, Hamburg, Germany). A total of three extraction steps were performed. The first extraction step contained 100 µM lactose, whereas the following extractions were performed using MCW extraction solution without lactose. The obtained extracts of each sample were pooled and 0.6 volumes of ddH_2_O were added to recover the soluble sugars in aqueous top phase.

#### Sample preparation and quantification

As recovered aqueous top phase may contain charged molecules which could interfere with ion chromatography (IC), collected top phase was pipetted through disposable chromatography column (BioRad, Hercules, CA, USA) containing cation exchange resin AG50W-X4 (BioRad), polyvinylpyrrolidone (PVPP, Acros Organics, Geel, Belgium), and anion exchange resin Amberlite IRA-67 free base (Merck, St. Louis, MO, USA) (Leach and Braun 2016). Briefly, 500 µL of AG50W-X4, 250 µL PVPP and 400 µL Amberlite IRA-67 free base slurries were added subsequently into disposable chromatography column. The resins were washed three times with ddH_2_O, before 1 mL of sample solution was added to remove strongly charged molecules. Finally, samples were taken up with 1 mL syringe (Merck) and filtered through 0.2 µm PVDF membrane (Roth, Karlsruhe, Germany). Samples were stored at − 20 °C, until they were sent for soluble sugar detection.

Carbohydrates were analyzed by IC coupled with pulsed amperometric detection on a Dionex™ ICS-5000 + System using an electrochemical detector with a gold electrode. Eluents were degassed by flushing with helium. An anion exchange 4 × 250-mm CarboPac PA1 column connected to a 4 × 50 mm guard column was used at 30 °C. The flow rate of the mobile phase was 1 mL min^−1^. A gradient with 150 mM NaOH (buffer A) and 0.5 M CH_3_COONa × 3 H_2_O in 150 mM NaOH (buffer B) was applied. Starting with 100% buffer A for 10 min then in 10 min to 50% buffer B; a wash step with 100% buffer B was applied for 5 min and subsequently 10 min 100% buffer A to equilibrate the column for the next sample (25 µL injection volume).

### RNA isolation and cDNA synthesis

RNA extraction was done using RNeasy Plant Mini Kit (Qiagen, Hilden, Germany). On-column DNA digestion was performed using RNase-Free DNase Set (Qiagen). All extraction steps were performed according to manufacturer’s instructions. RNA concentration and purity were assessed using Nano Drop 2000c (Thermo Fisher Scientific, Wilmington, DE, USA). Shoot samples were adjusted to 250 ng RNA µL^−1^ and directly colonized root samples to 100 ng RNA µL^−1^.

cDNA synthesis was done using Invitrogen SuperScript III reverse transcriptase (Thermo Fisher Scientific) according to manufacturer’s instructions. Briefly, 22 µL RNA, 2 µL dNTPs, 2 µL of hexa oligos and 2 µL of ddH_2_O were added together. The mixture was heated for 5 min at 65 °C followed by a 1 min incubation on ice. Subsequently, 8 µL 1st strand buffer, 2 µL 0.1 M DTT, 1 µL RiboLock RNase inhibitor (Thermo Fisher Scientific) and 1 µL reverse transcriptase was added to each sample followed by an incubation for 5 min at 25 °C. cDNA synthesis was performed for 60 min at 50 °C using a gradient Mastercycler (Eppendorf) followed by an inactivation step for 15 min at 75 °C. cDNA samples were stored at − 20 °C until further use.

### DNA isolation

Colonized plant roots were harvested at 10 dai. Subsequently, root material was washed to get rid of fungal mycelium attached to the root surface and stored at − 80 °C until further use. Samples were aliquoted (≤ 100 mg) and ground with a Mixer Mill MM 400 according to above-mentioned instructions. DNA extraction of colonized root material was performed using DNeasy Plant Mini Kit (Qiagen) according to manufacturer’s instructions. Samples were stored at − 20 °C until further use.

### Quantitative real-time PCR (qPCR)

qPCR was done using a peqSTAR 96Q Real-Time PCR Cycler (Peqlab Biotechnologie GmbH, Erlangen, Germany). Analysis was done using KAPA SYBR^®^ FAST qPCR Master Mix (2 ×) kit (Merck) containing KAPA SYBR FAST DNA polymerase, reaction buffer, dNTPs, SYBR Green I dye, and MgCl_2_ at a final concentration of 2.5 mM according to the manufacturer’s descriptions. A master mix with gene-specific primers was prepared for every gene containing 7 µL ddH_2_O, 10 µL KAPA SYBR^®^ FAST qPCR Master Mix (2 ×) kit, 0.5 µL of 10 mM 5′ primer and 0.5 µL of 10 mM 3′ primer. Eight genes coding for sucrose cleaving enzymes were analyzed: six sucrose synthases (*AtSUS1*, *AtSUS2*, *AtSUS3*, *AtSUS4*, *AtSUS5*, *AtSUS6*) and two cytosolic invertases (*AtCINV1*, *AtCINV2*). For plant defense, seven marker genes *AtEIN3*, *AtERF1*, *AtPDF1.2*, *AtOXI1*, *AtACS6*, *AtPR3,* and *AtBI1* were analyzed. Colonization rate of *S. indica* was assessed using specific primer pair for *SiTEF* gene. *AtUBP22* was used as an endogenous control according to Hofmann and Grundler (2007). All primer sequences are shown in the Supplementary Table S1. To each reaction well 2 µL of respective cDNA or DNA were added. A standard qPCR program consisted of an initial denaturation step (20 s at 95 °C) followed by 40 qPCR cycles (15 s at 95 °C and 20 s at 60 °C) and a melting stage.

The relative fold changes in gene expression were calculated based on the comparative CT method (Schmittgen and Livak 2008) using software peqSTAR96Q V2.1 (Peqlab Biotechnologie).

### Determination of growth parameters

*Arabidopsis thaliana* Col-0 plants as well as single mutant lines (*sus1-6* and *cinv1-2*) and multiple mutant lines (*sus1/2/3/4* and *cinv1/2*) were analyzed at 10 dai. Shoot fresh and dry weight was determined using an analytical balance model 770 (Kern & Sohn GmbH, Balingen, Germany). For analysis of dry shoot weight, shoots were harvested and dried for 3 h at 90 °C in a UF 260 plus (Memmert GmbH, Schwabach, Germany). Main root length was assessed with a ruler, and the number of side roots per main root length was calculated.

### Statistics

Statistical analysis was done using SPSS statistics software version 24.0 (Ehningen, Germany). qPCR-based gene expression study was performed in three independent biological repetitions (*n* = 3) with pooled plant material from 8–12 plants each. For colonization rate, qPCR tests were performed in eight independent biological repetitions (*n* = 8) with pooled plant material of 16–32 plants each. All measurements were performed with three technical replications. Differences in gene expression as compared to the respective control were assessed using Student’s *t* test (*P* < 0.05). Sugar pool analysis was performed in three independent repetitions (*n* = 3) with pooled plant material from 32–56 plants each. Differences between time points were assessed for each carbohydrate separately, using one-way ANOVA (Tukey’s test, *P* < 0.05). For fructose values in shoots of colonized plants, Welch ANOVA was performed (Dunnett-T3 test, *P* < 0.05). In the case of trehalose, due to missing data at 3 dai (2 of 3 measurements were below detection limit) the Student’s *t* test was performed (*P* < 0.05). Growth parameter experiments were performed in three independent biological repetitions with three samples each (*n* = 9). Each sample consisted of pooled plant material from three to ten plants. Outliers were removed with stem and leaf plot. Differences between colonized and noncolonized plants were assessed using two-way ANOVA (*P* < 0.05). When significant interaction was given, simple main effect analysis was performed (*P* < 0.01). In case of root length, variance of dependent variables was unequal (Levene test, *P* < 0.01) and the data of uncolonized *cinv1/2* were not normally distributed (Shapiro–Wilk, *P* < 0.05).

## Results

### *S. indica* changes hosts sugar pools

**Fig. 1:**
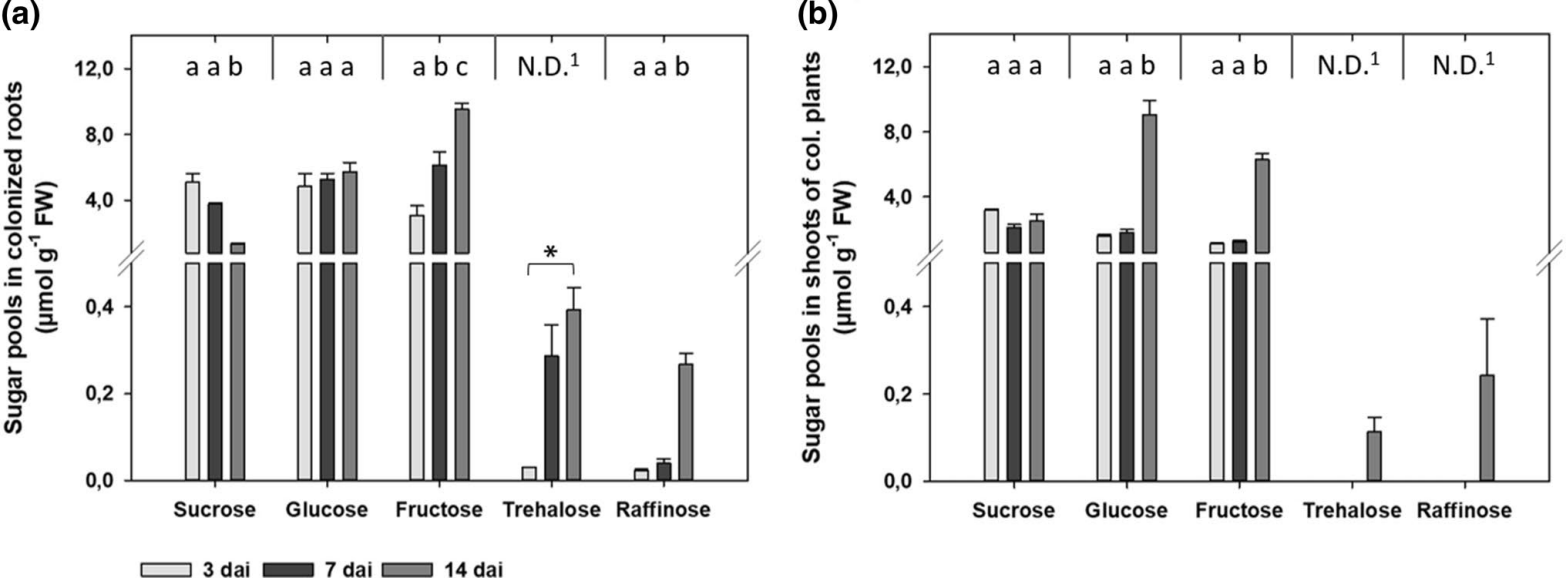
Sugar pool analysis of *A. thaliana* plants colonized by *S. indica*. **a** Sugar pools in colonized roots. **b** Sugar pools in shoots of colonized plants. Values indicate means ± SE of three independent repetitions from pooled plant material of 32–56 plantlets each. Means followed by a different letter indicate significant differences at *P* < 0.05 (Tukey’s test). In case of fructose values in shoots, Dunnett-T3 test was performed (*P* < 0.05). ^1^For trehalose and raffinose several samples were below detection limit of IC. In these cases, Student’s *t* test was performed, and asterisks indicate significant differences at *P* < 0.05

In colonized roots sucrose levels decreased significantly over time, showing lower values at 7 dai and 14 dai (Fig. 1a). In contrast, fructose values increased significantly, showing higher levels at 7 dai and 14 dai (Fig. 1a). For glucose no significant changes were observed.

Trehalose was only detectable in colonized roots, showing significant accumulation over time (Fig. 1a). At 3 dai it could only be detected in one of three biological replicates, as its amount was at detection limit. Similar to trehalose, raffinose accumulated significantly over time with higher values in roots colonized by *S. indica* (Fig. 1a).

Shoots of plants colonized by *S. indica* showed no significant differences in sucrose, glucose and fructose values during first two time points (Fig. 1b). At 14 dai, however, glucose and fructose values increased significantly (Fig. 1b). Trehalose and raffinose amounts were only detectable at 14 dai.

**Fig. 2:**
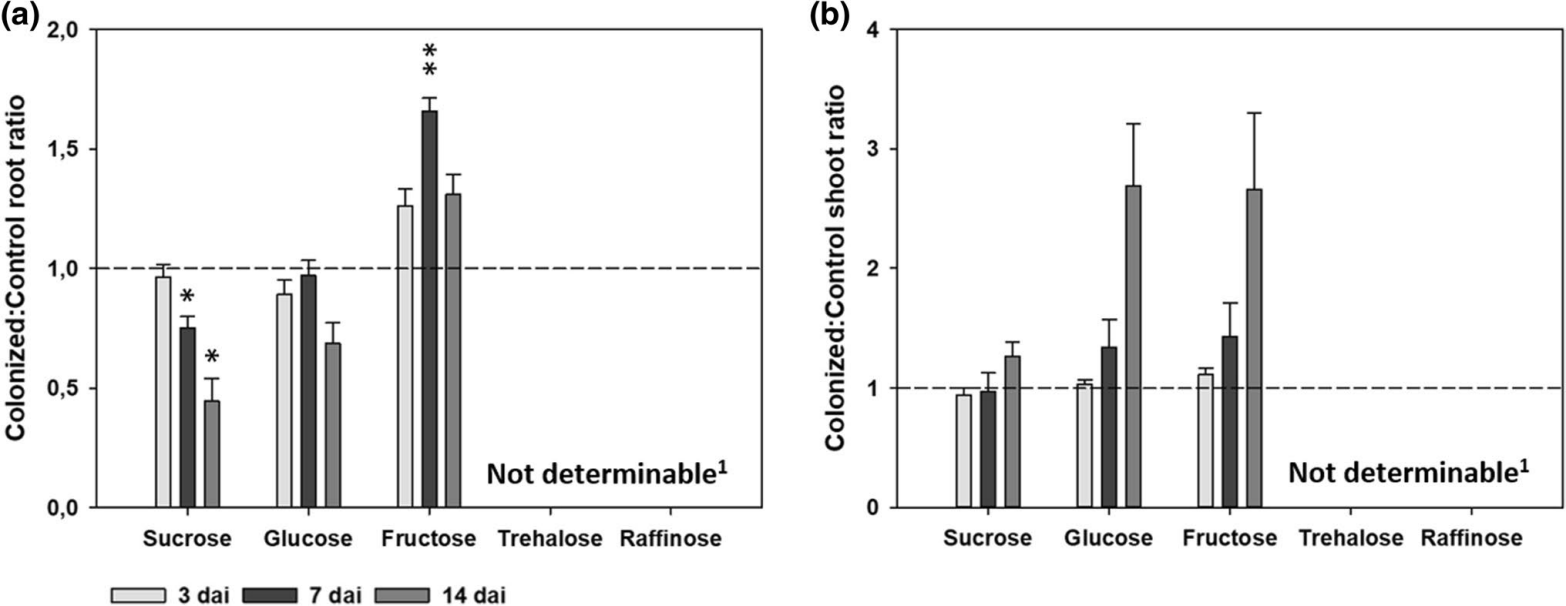
Sugar pool ratio of *A. thaliana* plants colonized by *S. indica* to noncolonized control plants. **a** Sugar pool ratio of colonized roots to control roots. **b** Sugar pool ratio of colonized shoots to control shoots. Values indicate means ± SE of three independent repetitions. Asterisks indicate significant differences between colonized and control samples (Student’s *t* test; *at *P* < 0.05; **at *P* < 0.01). ^1^Trehalose and raffinose ratio is not determinable as several samples were below detection limit of IC

To show differences between colonized and control samples, ratio of colonized to control samples was analyzed (Fig. 2). At 3 dai, no differences between colonized and noncolonized roots could be observed (Fig. 2a). In contrast, sucrose levels in colonized roots show significant lower values at 7 dai and 14 dai in comparison to noncolonized control roots (Fig. 2a).

Furthermore, fructose levels in colonized roots increased significantly over time showing higher values at 7 dai in comparison to their noncolonized controls (Fig. 2a). At 14 dai only a trend in upregulation was observed. Glucose levels were similar at first two time points and showed no significant decrease in colonized roots at 14 dai when compared with noncolonized roots (Fig. 2a). In shoots, at 3 and 7 dai the changes in levels of all tested sugars were not significant. At 14 dai, levels of glucose and fructose were increased, however, these changes were not significant in comparison to the noncolonized controls (Fig. 2b).

### *S. indica* modulates expression of hosts *AtSUS* and *AtCINV* genes

**Fig. 3:**
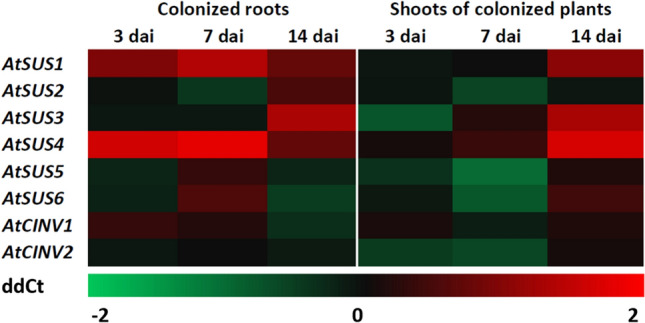
Gene expression of *AtSUS* and *AtCINV* genes in roots and shoots of *A. thaliana* plants colonized by *S. indica* in comparison to noncolonized controls at 3, 7 and 14 dai. Colors represent means of three biological independent repetitions. ddCt values are shown in Supplementary Table S2

To test whether there is a correlation between sugar pools and the expression of *AtSUS* and *AtCINV* genes in shoots and roots of colonized *A. thaliana* plants, qPCR analyses were performed. These results demonstrate different spatio-temporal patterns of those genes (Fig. 3 and Supplementary Table S2). In colonized roots, significant upregulation of sucrose synthase genes was found for *AtSUS4* at 3 dai and 7 dai, *AtSUS1* at 7 dai as well as *AtSUS3* at 14 dai. In shoots of colonized plants, significant downregulation of *AtSUS5* at 7 dai was found, whereas *AtSUS1* as well as *AtSUS3* showed significant upregulation at 14 dai. In general, the changes in *AtSUS* expression were only moderate, nevertheless clear trends could be observed. In roots at all time points moderate upregulation of majority of *AtSUSs* is visible, whereas in shoots the downregulation of several *AtSUSs* at 3 and 7 dai was observed. Time point 14 dai for shoots falls slightly out and does not match the trend observed for both earlier time points. *AtSUS1* and *AtSUS3* show at this time point significant upregulation. In contrast to *AtSUS*s, cytosolic invertases were not affected by *S. indica* colonization, neither in colonized roots, nor in shoots of colonized plants.

### *sus1/2/3/4* and *cinv1/2* do not exhibit* S. indica*-triggered growth promotion

To examine the importance of *AtSUS* and *AtCINV* genes for successful plant-*S. indica* interaction, growth parameters of *A. thaliana* mutant lines defective in multiple *AtSUS* and *AtCINV* upon *S. indica* colonization were analyzed. Preliminary tests of single mutants did not show significant changes when compared to the wild type (data not shown). In contrast, growth parameters in colonized *sus1/2/3/4* and *cinv1/2* showed distinctive changes (Table 2). In comparison to their noncolonized controls, both multiple mutant lines showed no changes in shoot fresh and dry weight whereas fresh and dry weight of colonized *A. thaliana* Col-0 in comparison to noncolonized control was significantly increased, 134% and 159%, respectively.

**Table 2:** Growth parameters of colonized and noncolonized *A. thaliana* plants 10 dai

	Col-0		*sus1/2/3/4*		*cinv1/2*	
	Control	*S. indica*	Control	*S. indica*	Control	*S. indica*
Shoot FW [mg]	6.21 (± 1.22)	**8.35 (± 0.87)**	5.43 (± 1.32)	5.62 (± 0.80)	4.02 (± 0.52)	4.19 (± 0.56)
Shoot DW [mg]	0.37 (± 0.09)	**0.59 (± 0.04)**	0.39 (± 0.09)	0.46 (± 0.04)	0.31 (± 0.02)	0.33 (± 0.02)
Main root length [mm]	52.4 (± 2.0)	**43.5 (± 3.5)**	46.7 (± 3.1)	**35.5 (± 3.3)**	9.2 (± 0.78)	9.2 (± 0.49)
Number of side roots per main root length	0.26 (± 0.04)	**0.38 (± 0.04)**	0.24 (± 0.04)	**0.33 (± 0.03)**	0.66 (± 0.06)	0.69 (± 0.04)

Values represent the mean ± SD of three biological independent repetitions with three samples each (*n* = 9). For each sample, plant material of three to 10 plants was pooled. Two-way ANOVA reveals significant interaction at *P* < 0.05. Bold values indicate significant difference according to simple main effect analysis at *P* < 0.01

The length of the main root was unaffected in *cinv1/2,* whereas *sus1/2/3/4* and *A. thaliana* Col-0 showed significant decrease, 76% and 83% respectively, when compared to the noncolonized controls (Table 2).

When concerning the number of side roots, no differences were found between colonized and noncolonized plants of line *cinv1/2*. In contrast, similar to the wild type the line *sus1/2/3/4* showed an increased number of side roots, when compared with the noncolonized controls, 146% and 138%, respectively (Table 2).

### Fungal colonization increased in *cinv1/2*

**Fig. 4:**
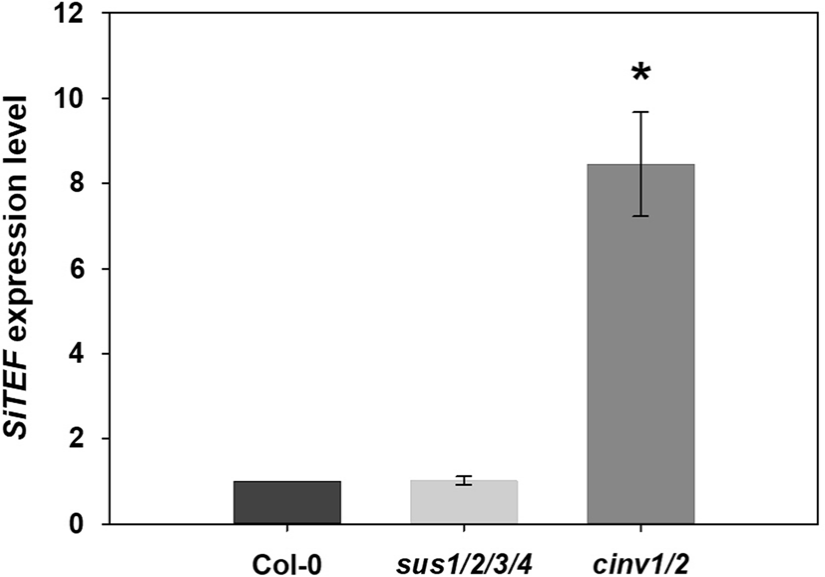
qPCR analysis of *SiTEF* abundance in colonized roots of *A. thaliana* Col-0 and mutant lines *sus1/2/3/4* and *cinv1/2* at 10 dai. Shown are means ± SE of eight independent biological repetitions. Endogenous control: *AtUBP22.* Asterisks indicate significant differences (Student’s *t* test, *P* < 0.001)

To reveal possible reasons for the lack of the typical growth promotion in *sus1/2/3/4* and *cinv1/2* lines upon *S. indica* colonization, we investigated the colonization rate in both mutants in comparison to colonized *A. thaliana* Col-0 using qPCR. *Serendipita indica* colonization was significantly increased in *cinv1/2*, whereas it remained unchanged in *sus1/2/3/4* in comparison to the wild type (Fig. 4).

### *S. indica* triggers changes in plant defense only in the wild type

**Fig. 5:**
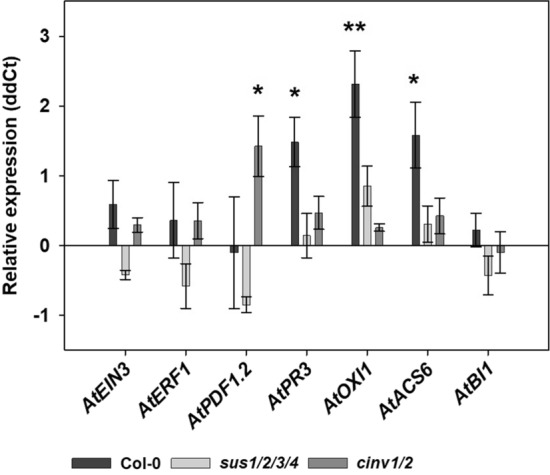
qPCR analysis of plant defense-related genes in colonized roots of *A. thaliana* Col-0 as well as mutant lines *sus1/2/3/4* and *cinv1/2* compared with roots of their noncolonized controls at 7 dai. Shown are the means ± SE of three independent biological repetitions. Endogenous control: *AtUBP22.* Asterisks indicate significant differences in between colonized and noncolonized control roots (Student’s *t* test; *at *P* < 0.05; **at *P* < 0.01)

To explain considerable differences in growth parameters and the colonization rate between mutant lines and the wild type we investigated several marker genes related to plant defense using qPCR. Significant upregulation of *AtPR3*, *AtOXI1* and *AtACS6* was observed in colonized *A. thaliana* wild type plants (Fig. 5). In contrast, *sus1/2/3/4* showed no significant changes in expression of either gene, whereas *cinv1/2* showed significant upregulation of *AtPDF1.2* (Fig. 5).

### Enhanced expression of defense genes in noncolonized *sus1/2/3/4* and *cinv1/2*

**Fig. 6:**
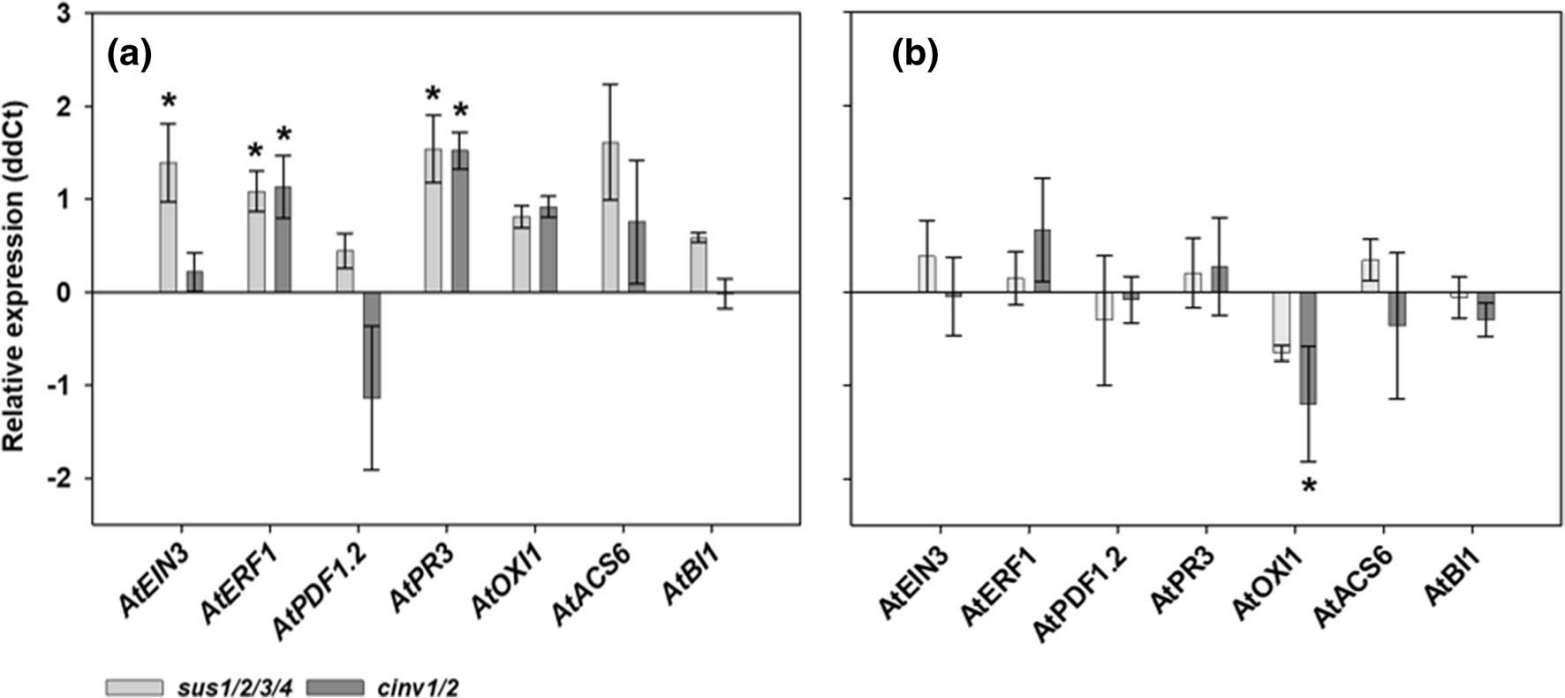
qPCR analysis of defense-related genes. **a** Noncolonized *sus1/2/3/4* and *cinv1/2* in comparison to uncolonized Col-0 at 7 dai. **b** Colonized *sus1/2/3/4* and *cinv1/2* in comparison to colonized Col-0 at 7 dai. Shown are the means ± SE of three independent biological repetitions. Endogenous control: *AtUBP22.* Asterisks indicate significant differences (Student’s *t* test, *P* < 0.05)

Noncolonized *sus1/2/3/4* and *cinv1/2* showed significant increase in expression of plant defense marker genes *AtERF1* and *AtPR3* when compared with noncolonized wild type. The expression of *AtEIN3* was significantly increased only in noncolonized *sus1/2/3/4* (Fig. 6a).

When gene expression of colonized *sus1/2/3/4* and *cinv1/2* was compared to colonized wild type, most of the genes showed no changes in their expression. Only *AtOXI1* was significantly downregulated in *cinv1/2* (Fig. 6b).

## Discussion

Although many recent studies have been delivering single puzzle stones to the whole picture, the root colonization process of *S. indica* and related molecular mechanisms are still not fully understood. In this work, we’ve focused on the sugar metabolism during the mutualistic interaction between this endophyte and *A. thaliana* and analyzed sugar shifts and the role of plant AtINVs and AtSUSs as well as coherent defense responses in more details. The measured sugar pools as well as expression patterns of *AtINV* and *AtSUS* genes in root and shoot tissue of colonized and control *A. thaliana* plants support the hypothesis that *S. indica* remotely controls host’s sugar-related processes to boost its own carbohydrate supply, which is mainly based on monosaccharides (Parniske 2008; Rani et al. 2016). The colonization of *A. thaliana* is divided in three main phases as suggested by Jacobs et al. (2011). Hence, to correlate our experimental set-up to these phases, plant material was harvested at 3, 7, and 14 dai corresponding to biotrophic phase, cell death-associated phase and intracellular sporulation phase, respectively. Changes in gene expression were rather moderate, which might be due to the lack of sucrose in the plant growing medium (Angeles-Núñez and Tiessen 2012; Wang and Ruan 2013). However, we observed general downregulation of genes coding for *AtSUS* and *AtCINV* at 3 dai and 7 dai in shoots that could lead to lower processing of sucrose. This sucrose excess subsequently gets transported to roots colonized by *S. indica*. Such enhanced sugar transport to the infection site being a strong sink was previously also shown for phylloxera in grapevine roots (Griesser et al. 2015). In our study, however, no significantly increased sucrose pools were found in shoot tissue of colonized *A. thaliana* plants, which is rather striking. To explain this phenomenon, Bezrutczyk et al. (2018) proposed that sugar fluxes and pool sizes do not necessarily correlate due to increased uptake by endophytic fungi or plant pathogens.

As shown here, the upregulated plant *AtSUS*s in root tissue would finally perform cleavage of transported sucrose into the monomers glucose and fructose. Interestingly, two *AtINV*s tested here did not show any changes in expression in roots at all time points. Thus, it will be necessary to analyze the expression of other members of this gene family. However, in other host plants colonized by *S. indica* such as soybean, the upregulation of several *INV*s was recently demonstrated (Bajaj et al. 2018). In rice colonized by another mutualistic fungus, *Phomopsis liquidambaris*, activation of soluble acid invertases was shown, which resulted in enhanced hexose fluxes in colonized roots positively influencing fungal colonization (Sun et al. 2020). In the case of *S. indica*, this activation might be additionally enhanced/facilitated by elicitors released by the fungus (Vadassery et al. 2009; Jisha et al. 2018). Not only plant invertases are involved in this process. It is known that fungi secrete their own invertases to increase monosaccharide level. The role of these enzymes in the sugar metabolisms during plant–endophyte interaction should not be underestimated. For instance, genes for invertase and phosphoglucose-isomerase were found in genomes of *S. indica* and *Serendipita herbamans* and the corresponding protein activities have been detected in liquid culture of both fungi (Vincenzo De Rocchis, personal communication). Furthermore, invertase domains are significantly expanded in the genome of *S. indica* (Lahrmann et al. 2013). We also could show increased expression of two invertases from *S. indica*, PIIN_03242 and PIIN_08245, in colonized *A. thaliana* roots at different time points (data will be published elsewhere).

**Fig. 7:**
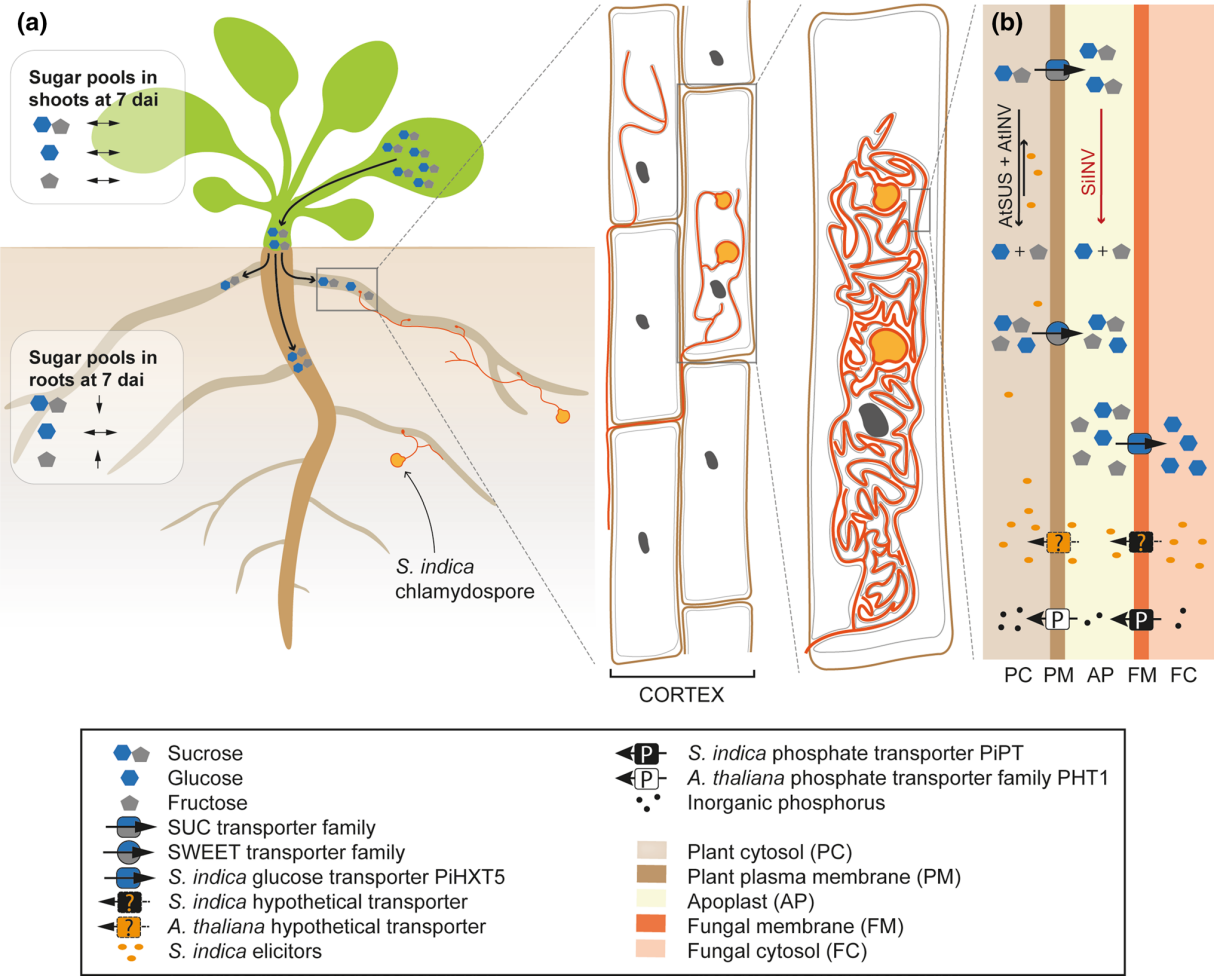
Putative sugar metabolism in *A. thaliana* during *S. indica* root colonization. **a** Colonization of root cortex cells via formation of intracellularly growing hypha. During this process coiled structures are formed, which ensure sophisticated interaction between *S. indica* and the host plant. In shoots, sugar pools are not affected by root *S. indica* colonization, however, increased flux of sucrose into roots might occur (Bezrutczyk et al. 2018). Interestingly, in *S. indica*-colonized roots only fructose amount increases significantly, whereas glucose level remains unchanged due to increased glucose uptake by *S. indica*. **b** Schematic overview of symbiotic interface formed during *S. indica* root colonization. In plant cytosol, there is increased cleavage of sucrose driven by plant invertases (AtINV) and sucrose synthases (AtSUS). This reaction might be enhanced/facilitated by elicitors released by *S. indica* (yellow dots; Vadassery et al. 2009; Jisha et al. 2018). In addition, in apoplast secreted fungal invertases (SiINV) lead to enhanced hexose level. Subsequently, plant SUC and SWEET transporters translocate available carbohydrates into apoplast, where remaining sucrose might be cleaved by invertases released by *S. indica* (red arrow, Vincenzo De Rocchis, personal communication; Lahrmann et al. 2013). Finally, glucose is taken up by *S. indica* selectively via e.g. high affinity transporter PiHXT5 (Rani et al. 2016). In exchange for this service, the root endophyte shifts inorganic phosphorus (black dots) into apoplast (Yadav et al. 2010), from where the host plant takes it up via members of phosphate transporter family PHT1 located in the plasma membrane (Nussaume et al. 2011). Adobe Illustrator was used to create the artwork

After the cleavage of sucrose by INVs and SUSs, monosaccharides can be taken up by *S. indica* using hexose transporters, whose high number was demonstrated in the *S. indica* genome (Zuccaro et al. 2009, 2011; Lahrmann et al. 2013). More recently, Rani et al. (2016) delivered a detailed functional characterization of *PiHXT5* and showed that it is regulated in response to mutualism as well as glucose concentration. Hexose withdrawal is also supported by the significant lower sucrose levels in colonized roots as shown here, which fits the *AtSUS*s expression patterns. At the same time, fructose levels are significantly increased, whereas glucose levels remain constant. As *PiHXT5* was indicated as high-affinity glucose transporter*,* and sucrose cleavage should lead to the formation of glucose and fructose in equimolar shares, measured carbohydrate pools support selective uptake of glucose. In shoots, downregulation of *AtSUS* and *AtCINV* at 3 and 7 dai was followed by upregulation at 14 dai. This might be due to the first signs of leaf chlorosis, although only green leaves were harvested for qPCR analysis. Nevertheless, the beginning of leaf senescence in those samples might be indicated by the upregulation of *AtSUS3* (Bieniawska et al. 2007), increased hexose levels (Wingler et al. 2006) and the presence of trehalose-6-phosphate (T6P, Wingler 2018). Furthermore, lack of sugar in the growing medium together with the simultaneous endophyte colonization, which speeds up the plant development, might have caused and/or increased this effect (Wingler and Roitsch 2008). To summarize our results and hints from available literature, the putative sugar metabolism, sugar–phosphorus exchange and involvement of elicitors in *S. indica*-colonized *A. thaliana* plants are shown in the Fig. 7.

Sucrose and its cleavage monosaccharides might not be only nutrients for *S. indica* but they might also play other roles in establishing the mutualistic relationship. There are several other aspects and functions of sucrose in plants that need to be taken into account (reviewed in Morkunas and Ratajczak 2014). For instance, sucrose functions as a hormone-like signaling molecule (Lou et al. 2007) and together with glucose and fructose induces sugar-specific changes in the gene expression (Kunz et al. 2014). This is the case in *S. indica*-colonized plants where downregulation of defense-related genes at early stages of colonization enables the plant–endophyte beneficial interaction (Schäfer et al. 2009; Camehl et al. 2011; Jacobs et al. 2011; Vahabi et al. 2015). In addition, UDP glucose is discussed to be an extracellular signaling molecule, which may even be perceived as damage-associated molecular pattern (Janse van Rensburg and Van den Ende 2017). Sucrose also plays a role in the induction of anthocyanin biosynthesis (Solfanelli et al. 2006; Das et al. 2012), which was shown in plants colonized by different fungal endophytes (Yu et al. 2020; Bu et al. 2020), and promotes phenylpropanoid biosynthesis (Ferri et al. 2011), which was shown in plants colonized by the endophyte *Gilmaniella *sp. (Yuan et al. 2019). T6P is both, a signal molecule and negative feedback regulator of sucrose in *A. thaliana* (Figueroa and Lunn 2016). There is a connection between trehalose formation and cell wall thickening as well as cell division (Gómez et al. 2006). Glucose has an important role as signal molecule interacting with CINV1 regulating *A. thaliana* root growth (Meng et al. 2020). Concerning all this, it is possible that at the early stage of the *S. indica*–host interaction two different processes take place. On the one hand, plant starts to defend itself, i.e., changing its sugar status that precisely modulates different defense responses (reviewed in Morkunas and Ratajczak 2014) and on the other hand *S. indica* actively manipulates the plant sugar levels in order to suppress these defense responses and to receive sufficient amounts of desired carbohydrates. This is in line with other studies showing a number of other plant pathogens or symbionts including some endophytic (e.g. *Glomus intraradices*; Schaarschmidt et al. 2007) and biotrophic fungi (e.g. *Blumeria gramini*; Scholes et al. 1994; Wright et al. 1995; Swarbrick et al. 2006), which change the sugar content of their hosts for their own benefit (reviewed in Tauzin and Giardina 2014).

We demonstrated that *S. indica* root colonization severely alters sugar pools and moderately changes the expression of *AtINV* and *AtSUS* genes in host tissues. In the next step we tested whether mutations in those genes have an impact on the host growth promotion triggered typically by *S. indica* colonization. *A. thaliana* Col-0 colonized by *S. indica* showed typical growth promoting effects, which is in line with available data (Peskan-Berghöfer et al. 2004). Similarly, we could see growth increase in single *inv* and *sus* mutants. Subsequently, we decided to test multiple mutants *cinv1/2* and *sus1/2/3/4*. According to Barratt et al. (2009), growth phenotype of the mutant line *cinv1/2* fits general carbon starvation due to reduced sucrose catabolism in roots. In comparison, mutant line *sus1/2/3/4* shows no differences in starch or sugar content and grows normally. In contrast to single mutant lines, in both multiple mutants, we did not detect any growth promotion, which might be due to disturbed sugar metabolism and signaling in both lines (reviewed in Bolouri Moghaddam and Van den Ende 2012; Trouvelot et al. 2014). This unsuccessful root endophyte–host interaction might be a result of nonfunctional diminishment of plant defense responses. Therefore, in the next step we analyzed the colonization rate of both multiple mutants. Interestingly, we found increased fungal colonization rate only in *cinv1/2* and not in *sus1/2/3/4*. This raises the question what might cause that? Barratt et al. (2009) demonstrated decreased invertase activity in *cinv1/2*, which might be the reason for altered *S. indica* root colonization. At least for AMF, Schaarschmidt et al. (2007) showed correlation between invertase activity in *Nicotiana tabacum* leaves and the degree of root mycorrhization by *Glomus intraradices*. For *S. indica* further research is required to shed more light on this phenomenon.

Another reason for enhanced colonization rate in *cinv1/2* could be altered defense responses in this mutant due to impaired sugar metabolism and signaling. Therefore, we tested expression of several defense-related genes in both mutants. Interestingly, when we compared colonized *sus1/2/3/4* and *cinv1/2* with their respective controls, the expression of these genes was mainly unaffected. This analysis however could not exclude if in noncolonized mutant lines defense was already altered prior to root endophyte colonization. Therefore, gene expression of noncolonized and colonized mutants was compared to uncolonized and colonized wild type plants, respectively. For the first combination we obtained increased expression of several defense-related genes (e.g. in *sus1/2/3/4 AtEIN3*, *AtERF1* and *AtPR3*; in *cinv1/2 AtERF1* and *AtPR3*). Thus, this finding strengthens the hypothesis that hampered processing of carbohydrates in these mutants have an impact on plant defense. In addition, comparing colonized wild type and colonized *sus1/2/3/4* and *cinv1/2* we did not observe much changes in gene expression. However, colonized roots of *cinv1/2* exhibited significant lower expression of *OXI1* (Oxidative signal inducible1) in comparison to the colonized wild type. Camehl et al. (2011) analyzed the role of OXI1 and PDK1 (3-phosphoinositide-dependent protein kinase1), a kinase that regulates OXI1, in growth promotion in response to *S. indica* colonization. The authors demonstrated that *OXI1* expression is upregulated in *S. indica*-colonized roots and that growth response is mediated by a cascade consisting of PDK1 and OXI1. Thus, significantly decreased *OXI1* expression in *cinv1/2* might be the reason for both, higher colonization rate and the lack of typical growth promotion. Moreover, this protein kinase regulates both basal resistance (pattern associated molecular patterns = PAMP) and effector triggered immunity (ETI) in response to the bacterial pathogen *Pseudomonas syringae* (Petersen et al. 2009). The authors reported on the importance of *OXI1* for immunity of *A. thaliana* against *P. syringae* and showed that both overexpression as well as down-regulation of *OXI1* can lead to a higher susceptibility against biotrophic pathogens. Hence, this finding delivers an additional possible explanation for increased accommodation of *S. indica* in *cinv1/2* roots. In roots of colonized *sus1/2/3/4* no downregulation of *OXI1* could be observed in comparison to the wild type. This explains similar colonization rate to the rate observed in the wild type roots.

Our results demonstrate that *S. indica* manipulates the sugar metabolism in order to change the sugar status as well as modulates the defense responses in *A. thaliana*. Thus, we postulate that in this interaction sugar is not only a nutrient for a fungus, but it also plays an important role in the regulation of defense responses, which is pivotal for the establishment of the mutualistic relationship. We showed that sugars are affecting the successful root colonization and further growth of *S. indica*, which might indicate the role of sugars as important signal molecules what has been recently postulated. This study shows that the interaction *A. thaliana*–*S. indica* is a balancing act between cooperation and exploitation, in which sugar metabolism plays a crucial role. Small changes in this mechanism can lead to severe disruption resulting in the lack of growth promotion or altered colonization rate.

### *Author contributions Statement*

MO: performed and conceived experiments, analyzed the data and designed illustrations; CL and RL: performed IC for sugar pool analysis; RD: conceived parts of the research; MO and KW: wrote the manuscript; SS: contributed with critical manuscript revisions; KW: conceived and designed the research. All authors read and approved the final manuscript.

## Supplementary Information

Below is the link to the electronic supplementary material.

Supplementary file1 (DOCX 19 KB)

## Abbreviations

AMF: Arbuscular mycorrhizal fungi
dai: Days after inoculation
(c)INV: (Cytosolic)invertase
SUS: Sucrose synthase
*sus*: *SUS* knock-down mutant
*inv*: *INV* knock-down mutant
