# Supplementary material for: Serendipita indica changes host sugar and defense status in Arabidopsis thaliana: cooperation or exploitation?
Source: Planta. 2021 Feb 23;253(3):74. doi: 10.1007/s00425-021-03587-3 (PMC7902589; doi:10.1007/s00425-021-03587-3)
Supplement: Supplementary file 1 — Supplementary file1 (DOCX 19 KB) [file 425_2021_3587_MOESM1_ESM.docx]

*Serendipita indica* changes host sugar and defense status in *Arabidopsis thaliana*: cooperation or exploitation?

Michael W Opitz^1^, Roshanak Daneshkhah^1^, Cindy Lorenz^2^, Roland Ludwig^2^, Siegrid Steinkellner^1^, Krzysztof Wieczorek^1^

^1^Institute of Plant Protection, Department of Crop Sciences, University of Natural Resources and Life Sciences, Tulln an der Donau, Austria

^2^Institute of Food Technology, Department of Food Sciences and Technology, University of Natural Resources and Life Sciences, Vienna, Austria

**Supplementary Table S1** Primer sequences used in this work

| **No.** | **Primer name** | **Sequence 5’—3’** | **Function** |
| --- | --- | --- | --- |
| 1 | *AtUBP22 F* | ACAACATATGACCCGTTTATCGA | Endogenous control |
| 2 | *AtUBP22 R* | TGTTTAGGCGGAACGGATACT |  |
| 3 | *AtSUS1 F* | GCGCGTCCACAGCCAACGTG | Sucrose synthase |
| 4 | *AtSUS1 R* | ACCAGGCCTTGGCCTCACAGC |  |
| 5 | *AtSUS2 F* | AGGGTGTACCAAATCTCAT | Sucrose synthase |
| 6 | *AtSUS2 R* | CATAGTGAAAGCTGTGTGG |  |
| 7 | *AtSUS3 F* | GAGCACGGGCTCTCGGGTTT | Sucrose synthase |
| 8 | *AtSUS3 R* | GCCGAGTCTCACGACGCTCC |  |
| 9 | *AtSUS4 F* | CACACTTCCCGGGTTGTACCGT | Sucrose synthase |
| 10 | *AtSUS4 R* | GCGCAAGCGAGTGTTCTTACCG |  |
| 11 | *AtSUS5 F* | GTCCTCGAAGCTCGGAGGGC | Sucrose synthase |
| 12 | *AtSUS5 R* | CTCCCTGCGCTTTCTCCCCA |  |
| 13 | *AtSUS6 F* | CGCCTTGATTGCAAGCCAGACC | Sucrose synthase |
| 14 | *AtSUS6 R* | TGGCCTGTCCTTGCTTCCTGC |  |
| 15 | *AtCINV1 F* | TCGAGGGCCATGAGTGGCGC | Cytosolic invertase |
| 16 | *AtCINV1 R* | CGCGCGTCTTGCGATCTGCG |  |
| 17 | *AtCINV2 F* | CGGTGGATCTTGGCCAGTATTGC | Cytosolic invertase |
| 18 | *AtCINV2 R* | CCAGCAATCTCGGTGTAGCCGT |  |
| 19 | *AtEIN3 F* | CATTTCTCCAGGTTACAATGAT | Transcription factor of AtERF1 |
| 20 | *AtEIN3 R* | AGCTTGTGGAACAGGAC |  |
| 21 | *AtERF1 F* | CGGCGGAGAGAGTTCAAGAGTC | Activator of AtPDF1.2 |
| 22 | *AtERF1 R* | TCCCACTATTTTCAGAAGACCCC |  |
| 23 | *AtPDF1.2 F* | CTGCTTTCGACGCACCGGCAA | Plant defense marker gene for JA/ET pathway |
| 24 | *AtPDF1.2 R* | ACCCCTGACCATGTCCCACTTGG |  |
| 25 | *AtOXI1 F* | TCATCTACATTGGCCGTGTC | Protein kinase required for activation of AtMPK6 |
| 26 | *AtOXI1 R* | CGTCGCTCCATACAACATCT |  |
| 27 | *AtACS6 F* | CCGGGAATGTTTGAAGTCTCTTG | Substrate for AtMPK6 |
| 28 | *AtACS6 R* | CGGTCTTAAGTCTGTGCACGG |  |
| 29 | *AtPR3 F* | ATCACCGCTGCAAAGTCCTTC | Pathogenesis related gene (chitinase) |
| 30 | *AtPR3 R* | TGCTGTAGCCCATCCACCTG |  |
| 31 | *AtBI1 F* | GCAGCAGCAATGTTAGCAAG | Attenuator of cell death |
| 32 | *AtBI1 R* | CACCACCATGTATCCCACAA |  |
| 33 | *SiTEF F* | ATCGTCGCTGTCAACAAGAT | Elongation factor 1 alpha |
| 34 | *SiTEF R* | ACCGTCTTGGGGTTGTATCC |  |

**Supplementary Table S2** Relative gene expression (ddCt) of *AtSUS* and *AtCINV* genes in roots and shoots of
*S. indica* colonized *A. thaliana* plants in comparison to non-colonized controls (*n*=3; ±SE). For each repetition, material from 8-12 plantlets was pooled. Black bold values indicate significant differences (student’s t-test, *P*<0.05)

| **genes** | **colonized roots** | | | **shoots of colonized plants** | | |
| --- | --- | --- | --- | --- | --- | --- |
|  | **3 dai** | **7 dai** | **14 dai** | **3 dai** | **7 dai** | **14 dai** |
| ***AtSUS1*** | 0.94 (±0.23) | **1.36 (±0.10)** | 0.72 (±0.58) | -0.10 (±0.14) | -0.02 (±0.31) | **1.03 (±0.22)** |
| ***AtSUS2*** | -0.06 (±0.18) | -0.44 (±0.24) | 0.50 (±0.17) | -0.09 (±0.05) | -0.59 (±0.09) | -0.10 (±0.27) |
| ***AtSUS3*** | -0.11 (±0.17) | -0.12 (±0.56) | **1.29 (±0.33)** | -0.78 (±0.14) | 0.20 (±0.20) | **1.27 (±0.33)** |
| ***AtSUS4*** | **1.61 (±0.39)** | **1.79 (±0.03)** | 0.71 (±0.68) | 0.07 (±0.14) | 0.36 (±0.76) | 1.65 (±0.93) |
| ***AtSUS5*** | -0.25 (±0.16) | 0.32 (±0.22) | -0.25 (±0.68) | -0.38 (±0.09) | **-1.01 (±0.29)** | 0.16 (±0.28) |
| ***AtSUS6*** | -0.22 (±0.13) | 0.53 (±0.12) | -0.52 (±0.26) | -0.12 (±0.27) | -0.80 (±0.37) | 0.42 (±0.25) |
| ***AtCINV1*** | 0.32 (±0.20) | 0.19 (±0.17) | -0.36 (±0.24) | 0.10 (±0.03) | -0.18 (±0.17) | 0.16 (±0.28) |
| ***AtCINV2*** | -0.11 (±0.08) | 0.00 (±0.09) | -0.15 (±0.33) | -0.49 (±0.31) | -0.61 (±0.08) | 0.07 (±0.22) |
